# Outcome of mechanical heart valve replacement in children: A 20-Year Experience

**DOI:** 10.1186/1749-8090-10-S1-A81

**Published:** 2015-12-16

**Authors:** Hong Ju Shin, Jo Won Jung, Han Ki Park, Young Hwan Park

**Affiliations:** 1 Severance Cardiovascular Hospital, 250 Seongsanno (134 Sinchon-dong), Seodaemun-gu, Seoul, Korea

## Background/Introduction

There are a few reports regarding outcome of mechanical valve replacement of children.

## Aims/Objectives

We investigated our 20-year experience with pediatric mechanical heart valve replacement with respect to mortality, valve-related morbidity, and reoperation risk factors.

## Method

The medical records of 42 patients (20 male, 47.6 %) who underwent 59 mechanical valve replacements between March 1992 and February 2014 were reviewed, retrospectively. Median age of the patients was 11.0 years (range, 9.1 months to 17.6 years) and 15 patients (25.4%) were less than 5 years. Congenital valve disease was the most common diagnosis (n = 16, 27.6%). Mitral valve replacement was performed in 39 patients, followed by aortic valve in 12 patients, pulmonic valve in five patients. Double valve replacements were performed in two patients. Mechanical valve was used in 51 cases (83.6%). The median valve size was 23 mm (range, 16 to 33 mm), and the median follow-up duration after valve replacement was 6.0 years (range, 15 days to 21.7 years). Events were defined as the following: thrombosis, embolism, bleeding, reoperation, and death.

## Results

There were two in-hospital mortality (all low cardiac outputs) and two late deaths (sepsis after heart transplantation and heart failure at 5.4 and 14.0 years postoperatively, respectively). Survival rates were 96.6%, 96.6%, and 93.7%, at 3, 5, and 10 years, respectively. Freedom from thromboembolism or bleeding events was 96.0 %, 93.9 %, and 86.0 %, at 3, 5 and 10years, respectively. Eighteen reoperations were performed postoperatively. Freedom from reoperation was 91.1 %, 88.9%, and 74.3%, at 3, 5, and 10years, respectively.

## Discussion/Conclusion

Mechanical valve replacement can be performed in children with favorable early and long-term survival. Thromboembolism or bleeding events due to anticoagulation therapy were not common.

